# Heteronemin Attenuates LPS-Induced Oxidative Stress and Inflammatory Responses in RAW264.7 Macrophages

**DOI:** 10.3390/md24100351

**Published:** 2026-10-06

**Authors:** Su-Jin Lee, Long Hoang To, Chulhong Oh, Gun-Hoo Park, Yeon-Ju Lee

**Affiliations:** 1 Korea Institute of Ocean Science and Technology, 2670, Iljudong-ro, Gujwa-eup, Jeju-si 63349, Republic of Korea; sujinlee5699@gmail.com (S.-J.L.); och0101@kiost.ac.kr (C.O.); 2 Korea Institute of Ocean Science and Technology, 385, Haeyang-ro, Yeongdo-gu, Busan Metropolitan City 49111, Republic of Korea; longhto@kiost.ac.kr; 3 Department of Marine Technology and Convergence Engineering, University of Science and Technology, Daejeon 34113, Republic of Korea

**Keywords:** heteronemin, scalarane sesterterpenoid, anti-inflammatory activity, RAW264.7 macrophages, ROS, iNOS, COX-2, MAPK signaling

## Abstract

Marine-derived natural products have attracted considerable attention as potential anti-inflammatory agents. Although the anticancer and pro-oxidative effects of heteronemin, a marine sponge-derived scalarane-type sesterterpenoid, have been reported, its direct regulatory effects on inflammatory mediator production in activated macrophages remain unclear. In this study, we aimed to investigate the anti-inflammatory effects of heteronemin in lipopolysaccharide (LPS)-stimulated RAW264.7 macrophages. Heteronemin significantly suppressed LPS-induced nitric oxide production at concentrations up to 10 μM with minimal effects on cell viability. In addition, heteronemin reduced the expressions of inducible nitric oxide synthase (iNOS) and cyclooxygenase-2 (COX-2), attenuated intracellular reactive oxygen species (ROS) accumulation, and downregulated pro-inflammatory cytokines, including tumor necrosis factor-alpha (TNF-α), interleukin-1 beta (IL-1β), and interleukin-6 (IL-6). Furthermore, heteronemin treatment was associated with reduced phosphorylation of mitogen-activated protein kinases (MAPKs), including p38, JNK, and ERK, following prolonged LPS stimulation. In contrast to its previously reported ROS-inducing activity in cancer cells, heteronemin reduced inflammation-associated ROS accumulation and was associated with decreased MAPK phosphorylation in LPS-stimulated macrophages, suggesting that its effects on cellular redox responses may depend on the experimental context. Collectively, these findings suggest the therapeutic potential of heteronemin as a promising marine-derived lead compound for inflammatory diseases.

## 1. Introduction

Chronic inflammation is a major pathological factor contributing to the progression of numerous diseases, including metabolic disorders, cardiovascular diseases, neurodegenerative diseases, and inflammatory conditions [1,2]. Persistent inflammatory responses are characterized by the excessive production of inflammatory mediators and pro-inflammatory cytokines, leading to intracellular reactive oxygen species (ROS) accumulation and activation of inflammatory signaling pathways, including mitogen-activated protein kinase (MAPK) signaling [3,4]. Activated macrophages play a central role in these processes through the production of nitric oxide (NO), cytokines, and inflammatory enzymes such as inducible nitric oxide synthase (iNOS) and cyclooxygenase-2 (COX-2) [5].

Although anti-inflammatory drugs and biological therapies are widely used clinically, their long-term use is often limited by adverse effects, insufficient efficacy, and high cost [1]. Therefore, the development of novel therapeutic agents capable of modulating inflammatory signaling pathways remains an important therapeutic objective [6,7]. In this regard, marine-derived natural products have attracted increasing attention as promising sources of structurally diverse bioactive molecules with pharmaceutical potential [8,9]. In particular, marine sponge-derived terpenoids have demonstrated diverse biological activities, including antimicrobial, anticancer, and anti-inflammatory effects [10,11,12,13,14].

Scalarane sesterterpenoids are a characteristic subclass of marine sponge-derived terpenoids possessing a rigid pentacyclic C25 carbon skeleton [9]. Scalarane-type sesterterpenoids exhibit diverse biological activities, including cytotoxic, antimicrobial, antitubercular, and anti-inflammatory properties [10,14,15,16]. Among these compounds, heteronemin, isolated from marine sponges of the genera *Hyrtios* and *Hippospongia*, has been extensively investigated for its anticancer potential [17,18]. Heteronemin has been reported to induce intracellular ROS accumulation, mitochondrial dysfunction, and apoptotic cell death in various cancer cell lines. In addition, previous studies have suggested that heteronemin modulates the NF-κB- and MAPK-associated pathways in cancer-related cellular contexts [12,19].

Despite increasing evidence regarding the anticancer potential of heteronemin, its effects on inflammatory responses in activated macrophages remain poorly understood. In particular, whether heteronemin modulates LPS-induced inflammatory responses, including NO and pro-inflammatory cytokine production, inflammatory mediator expression, and intracellular ROS accumulation, has not been fully elucidated. Given the previously reported ROS-inducing activity of heteronemin in cancer cells, its role in regulating inflammation-associated oxidative stress in activated macrophages warrants further investigation.

Therefore, in the present study, we investigated the anti-inflammatory effects of heteronemin using LPS-stimulated RAW264.7 macrophages as an in vitro inflammatory model. Specifically, we examined its effects on NO production, inflammatory mediator expression, pro-inflammatory cytokine production, intracellular ROS accumulation, and MAPK-associated signaling pathways under conditions that did not markedly affect macrophage viability. By addressing the largely unexplored anti-inflammatory activity of heteronemin in activated macrophages, this study extends heteronemin’s biological relevance beyond the predominantly anticancer activities characterized in previous studies and provides new insights into its potential role in regulating macrophage-mediated inflammatory and oxidative stress responses.

## 2. Results

### 2.1. Effect of Heteronemin on Cell Viability

**Figure 1:**
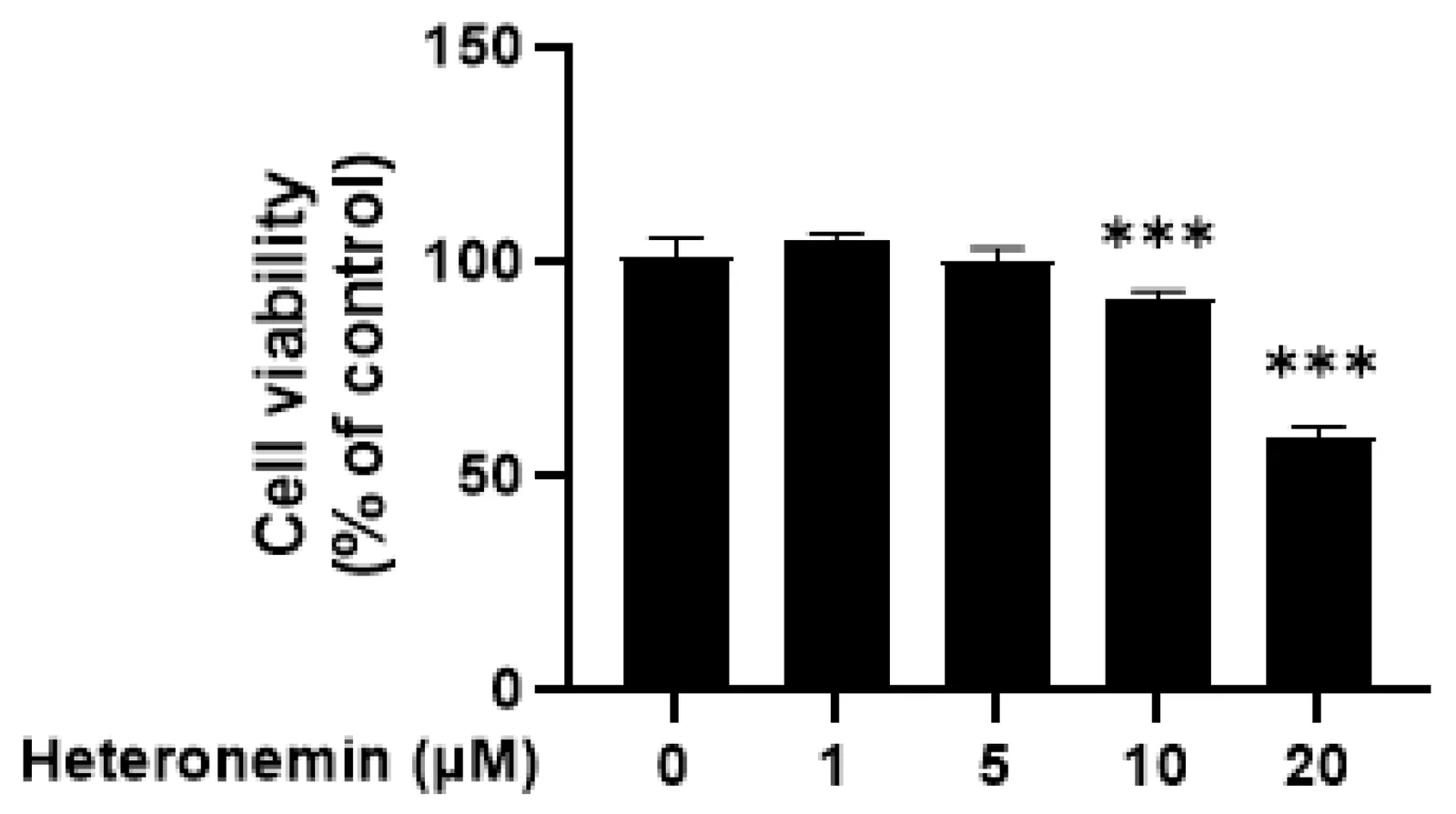
Effect of heteronemin on cell viability. RAW264.7 cells were treated with the indicated concentrations of heteronemin (0–20 μM) for 24 h, and cell viability was quantified using the WST-8 assay. Data are expressed as the mean ± standard deviation (SD) of three independent experiments performed in triplicate. *** *p* < 0.001 vs. control.

Heteronemin was evaluated for its biological activity in RAW264.7 macrophages. To determine the non-cytotoxic concentration range prior to subsequent anti-inflammatory experiments, RAW264.7 cells were treated with heteronemin (0–20 μM) for 24 h, and cell viability was assessed using the WST-8 assay. Cell survival remained high at concentrations up to 10 μM (approximately 95% relative to that of the control), indicating that heteronemin did not exert biologically relevant cytotoxic effects up to this concentration (Figure 1). In contrast, treatment with 20 μM heteronemin markedly reduced cell viability to approximately 59.1%, suggesting dose-dependent cytotoxic effects at higher concentrations.

### 2.2. Inhibition of LPS-Induced NO Production by Heteronemin

**Figure 2:**
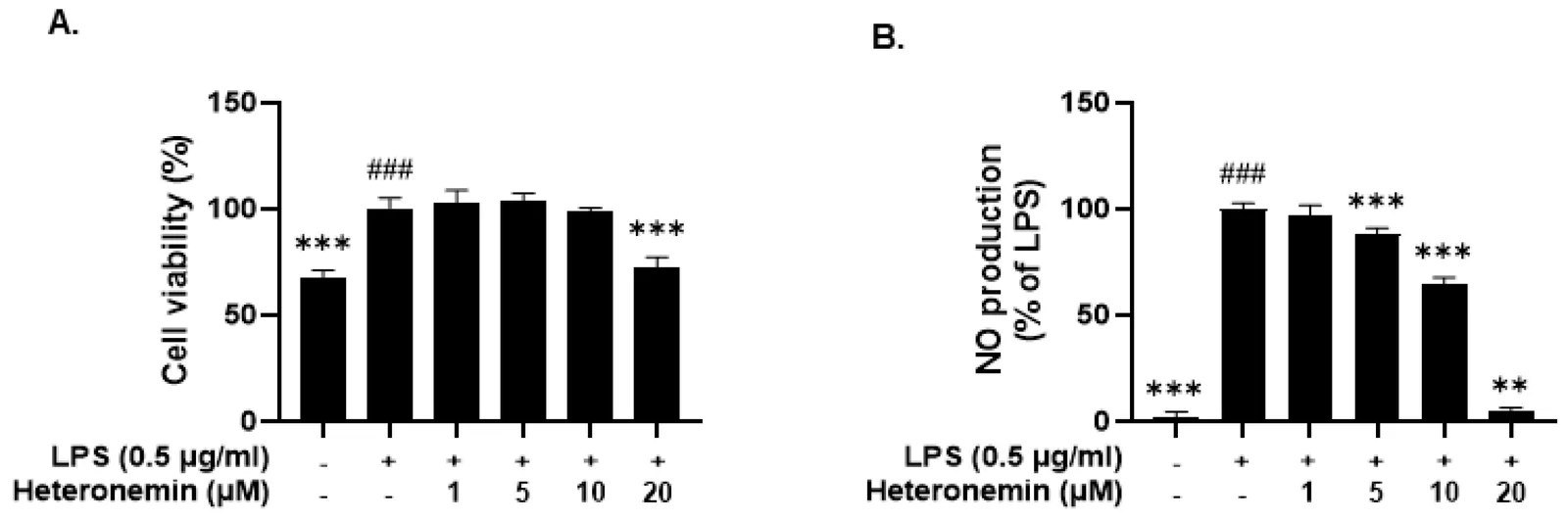
Heteronemin suppresses lipopolysaccharide (LPS)−induced nitric oxide (NO) production in RAW264.7 macrophages. RAW264.7 macrophages were pre-treated with heteronemin at the indicated concentrations for 2 h, followed by stimulation with LPS (0.5 μg/mL) for 24 h. (**A**) Cell viability was determined using the WST-8 assay to confirm non-cytotoxicity under inflammatory conditions. (**B**) NO levels in the culture supernatants were quantified using the Griess assay. Data are expressed as the mean ± SD of three independent experiments performed in triplicate. ** *p* < 0.01, *** *p* < 0.001 vs. LPS−treated group; ^###^ *p* < 0.001 vs. untreated control.

To evaluate the anti-inflammatory activity, RAW264.7 macrophages were pretreated with heteronemin for 2 h prior to LPS stimulation (0.5 μg/mL) for 24 h. Cell viability was maintained at concentrations of up to 10 μM, whereas treatment with 20 μM heteronemin reduced cell viability by approximately 30% (Figure 2A).

Under these conditions, heteronemin suppressed LPS-induced NO production in a concentration-dependent manner (Figure 2B). NO levels were minimally affected at lower concentrations (1–5 μM) but were significantly reduced at 10 μM, while near-complete inhibition was observed at 20 μM. Since 20 μM heteronemin was cytotoxic, this concentration was excluded from subsequent experiments. Therefore, 10 μM was selected as the highest non-cytotoxic concentration for further anti-inflammatory analyses.

### 2.3. Inhibitory Effect of Heteronemin on LPS-Induced Oxidative Stress in RAW264.7 Macrophages

**Figure 3:**
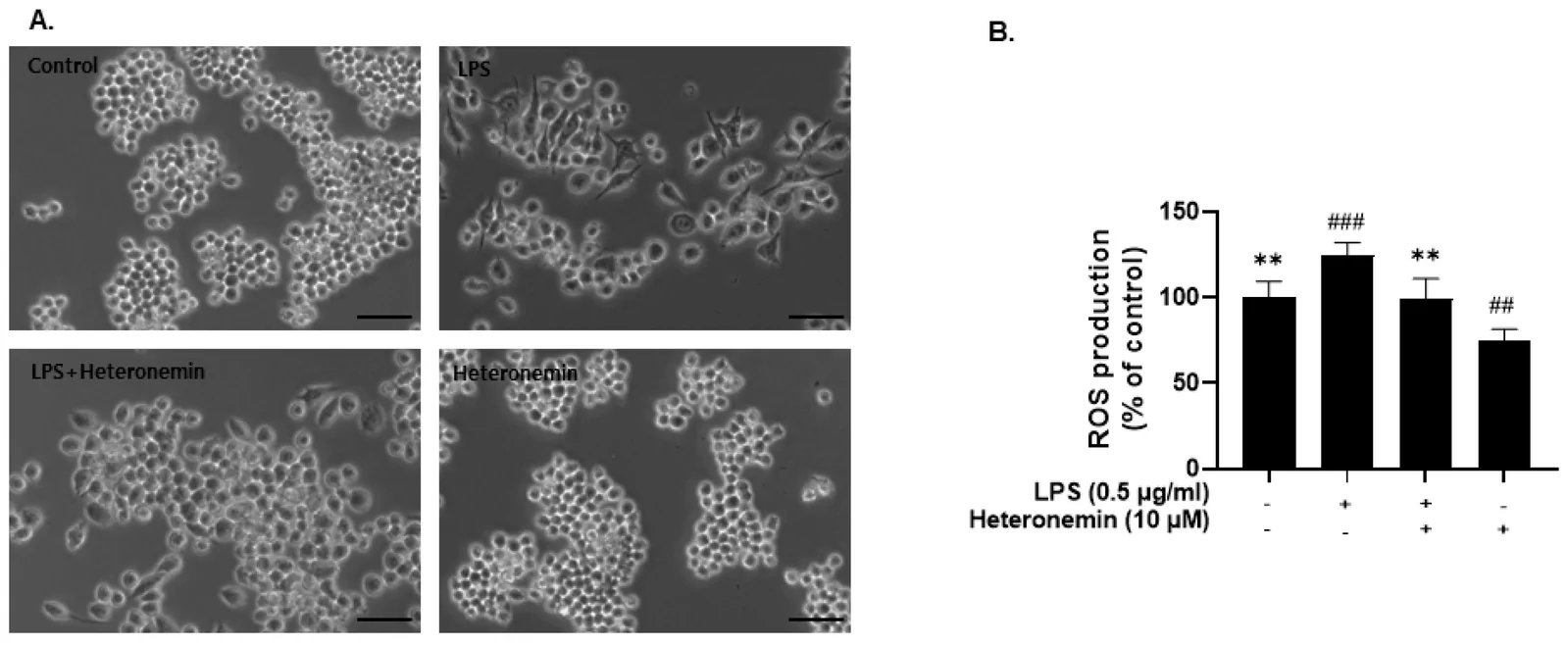
Effects of heteronemin on LPS−induced reactive oxygen species (ROS) production in RAW264.7 cells. (**A**) Representative optical microscopic images of RAW264.7 cells under different treatment conditions: Control, LPS, LPS + heteronemin, and heteronemin alone. LPS treatment induced noticeable morphological changes in RAW264.7 cells, whereas co-treatment with heteronemin attenuated these alterations. Scale bar = 100 μm. (**B**) Intracellular ROS levels in RAW264.7 cells. Cells were pretreated with heteronemin (10 μM) for 2 h and subsequently stimulated with LPS (0.5 µg/mL) for 24 h. Intracellular ROS levels were measured using the H_2_DCFDA assay and expressed as a percentage of the untreated control. Data are presented as the mean ± SD from three independent experiments. ^###^ *p* < 0.001, ^##^ *p* < 0.01 vs. untreated control; ** *p* < 0.01 vs. LPS-treated group.

LPS stimulation induced remarkable morphological changes in RAW264.7 macrophages, including increased cell spreading and an activated cellular appearance, whereas co-treatment with heteronemin attenuated these LPS-induced morphological alterations. The morphology of cells treated with heteronemin alone was comparable to that of the untreated cells of the control group (Figure 3A).

To further investigate whether these morphological changes were associated with intracellular oxidative stress, overall ROS levels were measured using the fluorescent probe H_2_DCFDA (Figure 3B). LPS stimulation increased intracellular ROS levels by approximately 24.6% compared with the control group, whereas co-treatment with heteronemin attenuated this increase, reducing ROS levels to 99.4% of the control level. Notably, heteronemin treatment alone decreased basal intracellular ROS levels by approximately 24.9% compared with the control group.

### 2.4. Heteronemin Suppresses LPS-Induced Pro-Inflammatory Cytokine Expression in RAW264.7 Macrophages

**Figure 4:**
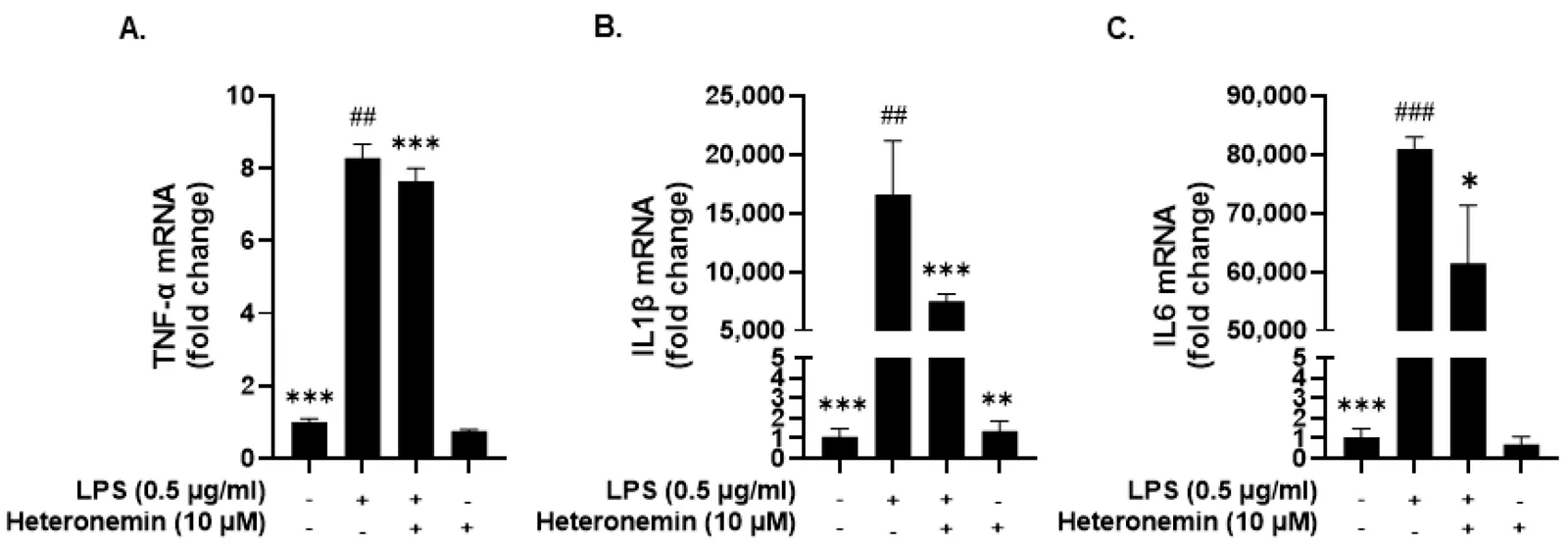
Anti-inflammatory effects of heteronemin on LPS−induced pro-inflammatory cytokine expression in RAW264.7 macrophages. RAW264.7 cells were pre-treated with heteronemin for 2 h followed by stimulation with LPS (0.5 μg/mL) for 24 h. The mRNA expression levels of (**A**) TNF−α, (**B**) IL−1β and (**C**) IL−6 were quantified using quantitative real-time PCR and normalized to the control group. Data are presented as the mean ± SD. ^##^ *p* < 0.01, ^###^ *p* < 0.001 vs. untreated control; * *p* < 0.05, ** *p* < 0.01, *** *p* < 0.001 vs. LPS-treated group.

The mRNA expression levels of LPS-inducible cytokines (tumor necrosis factor-alpha (TNF-α), interleukin-1 beta (IL-1β), and interleukin-6 (IL-6)) in RAW264.7 macrophages were quantified using qRT-PCR. LPS stimulation markedly increased the expression of all three cytokines relative to those of the control group (Figure 4). In the presence of heteronemin, LPS-induced cytokine expression was reduced to varying extents, with approximate decreases of 8, 55, and 24% in the expression of TNF-α, IL-1β and IL-6, respectively, relative to those of the LPS-only group (Figure 4). In contrast, treatment with heteronemin alone did not alter basal cytokine expression, and mRNA levels remained comparable to those observed in the control group.

### 2.5. Heteronemin Suppresses COX-2 and iNOS Expression at the mRNA and Protein Levels

**Figure 5:**
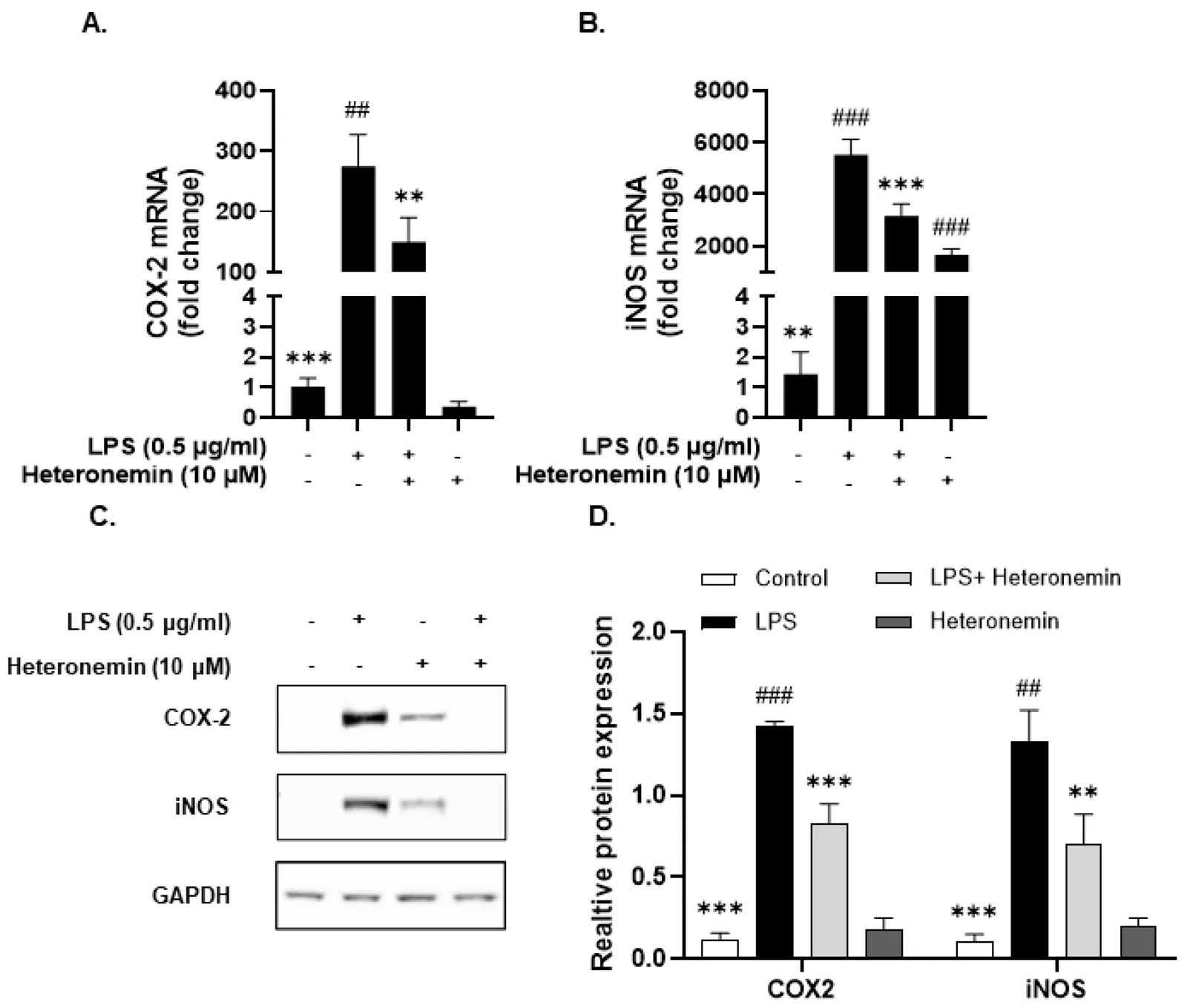
Heteronemin attenuates LPS−induced COX−2 and iNOS expression. Quantitative PCR analysis of (**A**) COX−2 and (**B**) iNOS mRNA levels in RAW264.7 macrophages. Cells were pretreated with 10 µM heteronemin for 2 h, followed by stimulation with LPS (0.5 µg/mL) for 24 h. Gene expression levels were normalized to those of GAPDH and are presented as fold changes relative to the untreated control. (**C**) Representative Western blot images showing COX−2, iNOS, and GAPDH protein expression under the same treatment conditions. (**D**) Band intensities were quantified using densitometry and normalized to that of GAPDH. Data are presented as the mean ± SD. ^##^ *p* < 0.01, ^###^ *p* < 0.001 vs. untreated control; ** *p* < 0.01, *** *p* < 0.001 vs. LPS-treated group.

To further evaluate the effect of heteronemin on LPS-inducible inflammatory enzymes, we examined its impact on COX-2 and iNOS expression in RAW264.7 macrophages. Under control conditions, both genes exhibited low basal expression at the transcriptional and translational levels. LPS stimulation markedly increased COX-2 and iNOS mRNA expression, whereas co-treatment with heteronemin significantly attenuated this induction (Figure 5A,B). Compared with the control group, heteronemin treatment alone slightly decreased COX-2 mRNA expression, though the difference was not statistically significant (Figure 5A), whereas it significantly increased iNOS mRNA expression (^###^ *p* < 0.001; Figure 5B).

Consistent with the transcriptional data, protein expression analysis demonstrated that LPS markedly increased COX-2 and iNOS protein levels, which were reduced by co-treatment with heteronemin (Figure 5C,D). However, heteronemin alone did not induce detectable changes in COX-2 or iNOS protein expression, maintaining levels comparable to the basal conditions (Figure 5C,D).

### 2.6. Heteronemin Suppresses LPS-Induced MAPK Activation

**Figure 6:**
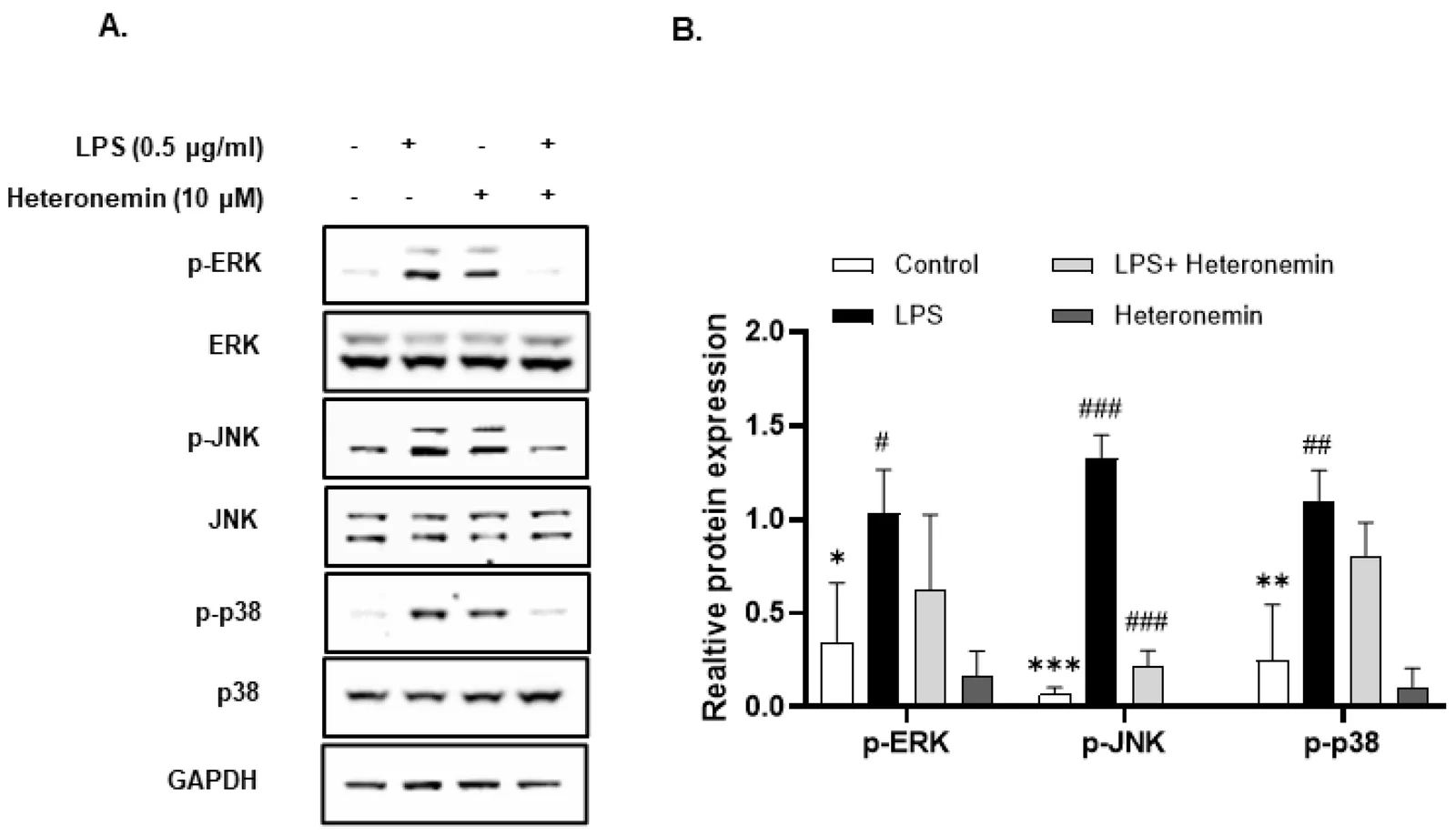
Heteronemin suppresses LPS−induced MAPK activation in RAW264.7 macrophages. (**A**) Representative Western blot images showing the phosphorylation levels of ERK, JNK, and p38 MAPKs in RAW264.7 macrophages pretreated with heteronemin (10 µM) for 2 h followed by stimulation with LPS (0.5 µg/mL) for 24 h. Total ERK, JNK, and p38 protein levels were analyzed as controls for MAPK activation, and GAPDH was used as a loading control. (**B**) Band intensities of phosphorylated ERK, JNK, and p38 were quantified via densitometric analysis and normalized to their corresponding total protein levels. Data are presented as the mean ± SD from three independent experiments. ^#^ *p* < 0.05, ^##^ *p* < 0.01, ^###^ *p* < 0.001 vs. untreated control; * *p* < 0.05, ** *p* < 0.01, *** *p* < 0.001 vs. LPS-treated group.

To further investigate the effect of heteronemin on intracellular signaling pathways under inflammatory conditions, the activation status of the MAPK family members p38, JNK, and ERK was examined in LPS-stimulated RAW264.7 macrophages. The cells were pretreated with heteronemin prior to LPS exposure, and MAPK activation was assessed by analyzing the phosphorylation levels of each kinase. The phosphorylation of p38, JNK, and ERK markedly increased upon LPS stimulation compared with that in the control group. In contrast, pretreatment with heteronemin attenuated the LPS-induced phosphorylation of all three MAPKs (Figure 6). Importantly, the total protein levels of p38, JNK, and ERK were not altered by either LPS or heteronemin treatment and remained comparable across all experimental groups, indicating that the observed changes can be attributed to differences in phosphorylation status rather than changes in overall protein expression.

## 3. Discussion

In the present study, heteronemin attenuated LPS-induced inflammatory responses in RAW264.7 macrophages, providing direct evidence of its anti-inflammatory activity. Previous studies have primarily focused on the anticancer effects of heteronemin in cancer cell models, including inhibition of TNFα-induced NF-κB activation and induction of apoptosis [19]. Heteronemin reduced NO production at 10 μM without affecting cell viability, whereas 20 μM heteronemin reduced both NO production and cell viability. Thus, the anti-inflammatory activity of heteronemin should be interpreted with consideration of its effects on cell viability. Further studies using a broader range of concentrations are warranted to better define the relationship between its anti-inflammatory activity and cytotoxicity.

LPS is a well-established stimulus that promotes a pro-inflammatory M1-like phenotype in macrophages, characterized by increased expression of iNOS and production of NO and pro-inflammatory cytokines [20]. In contrast, M2-like macrophages are generally associated with anti-inflammatory and tissue-repair functions and are typically induced by stimuli such as IL-4 and IL-13 [21]. Accordingly, the LPS-stimulated RAW264.7 model used in the present study primarily reflects M1-like inflammatory activation. However, because M2-associated markers were not assessed, the effects of heteronemin observed in this study should be interpreted as suppression of LPS-induced pro-inflammatory activation rather than evidence of M1-to-M2 repolarization.

At the non-cytotoxic concentration tested, heteronemin reduced NO production and the expression of iNOS and COX-2, as well as the mRNA levels of the pro-inflammatory cytokines TNF-α, IL-1β, and IL-6. The reduction in NO production accompanied by decreased iNOS expression suggests that modulation of the iNOS/NO axis may contribute to the anti-inflammatory activity of heteronemin. Furthermore, the decreased expression of COX-2 and multiple pro-inflammatory cytokines suggests that heteronemin suppresses several components of the inflammatory response. The reduction in TNF-α mRNA expression was modest compared with those observed for IL-1β and IL-6, suggesting that the effects of heteronemin may vary among individual inflammatory cytokines; however, the mechanisms underlying these differential responses remain unclear.

A notable finding of the present study was the reduction in intracellular ROS accumulation following heteronemin treatment, accompanied by reduced phosphorylation of p38, JNK, and ERK under prolonged LPS stimulation. Given that excessive ROS can amplify inflammatory responses through redox-sensitive signaling pathways, including MAPKs [22], the concomitant reductions in intracellular ROS and MAPK phosphorylation may reflect attenuation of the inflammatory response.

However, the present study did not establish a causal relationship between ROS reduction and MAPK phosphorylation. Moreover, MAPK phosphorylation is a rapid and dynamic signaling event, whereas phosphorylation levels in the present study were assessed after 2 h of heteronemin pretreatment followed by 24 h of LPS stimulation. Therefore, these findings should not be interpreted as evidence that heteronemin directly or acutely inhibits MAPK activation, but rather as an association observed under prolonged LPS stimulation.

A previous study in A498 human renal carcinoma cells reported time-dependent changes in MAPK phosphorylation following heteronemin treatment (3 μM), including transient increases in ERK and JNK phosphorylation within 15 min, increased p38 phosphorylation at 30 min, and sustained suppression of ERK phosphorylation from 1 to 24 h [17]. Although these findings demonstrate that heteronemin can induce both early and prolonged changes in MAPK signaling, their relevance to LPS-stimulated macrophages remains unclear because of differences in cellular context and inflammatory stimulation. In the present study, the reduced MAPK phosphorylation observed after prolonged LPS exposure may reflect changes in the inflammatory signaling state. However, whether these changes are mediated by reduced ROS production or other secondary cellular responses remains to be determined.

Furthermore, the stability and half-life of heteronemin under the present cell culture conditions remain unknown. Future studies assessing early MAPK phosphorylation kinetics and the stability of heteronemin during LPS stimulation, together with experiments using antioxidants such as N-acetylcysteine (NAC) and selective MAPK inhibitors, are warranted to clarify the temporal effects of heteronemin on MAPK signaling and the potential involvement of ROS modulation.

The methodology used to assess intracellular ROS should also be considered in interpreting these findings. H_2_DCFDA is useful for detecting changes in intracellular oxidative status but does not permit identification of specific ROS or their intracellular sources [23]. Thus, the present study cannot determine whether the observed reduction in ROS primarily reflects changes in mitochondrial ROS, NADPH oxidase-derived ROS, or other intracellular sources. The use of source-specific probes and complementary analytical approaches will be necessary to further characterize the intracellular sources underlying the ROS-modulating effects of heteronemin.

Interestingly, the reduction in intracellular ROS observed in the present study contrasts with previous reports describing heteronemin as a pro-oxidative compound in cancer cells [11,18]. In hepatocellular carcinoma cells, heteronemin-induced ROS accumulation was associated with ERK downregulation and the induction of apoptosis and ferroptosis through oxidative stress-related mechanisms [12]. In prostate and renal carcinoma cells, heteronemin has also been reported to induce mitochondrial dysfunction, endoplasmic reticulum (ER) stress, caspase activation, and stress-associated MAPK signaling, ultimately promoting apoptotic cell death [17]. These previous findings were obtained in cancer cells under treatment conditions that differed from those used in the present LPS-stimulated macrophage model, particularly with respect to cell type, heteronemin concentration, and exposure duration. Therefore, the contrasting ROS responses observed across studies may reflect context-dependent effects of heteronemin on cellular redox homeostasis rather than a uniform pro-oxidative or antioxidative action.

Although NF-κB is a key pathway involved in macrophage-mediated inflammatory responses [24,25], NF-κB activation and upstream TLR4-associated signaling were not directly assessed in the present study. Further studies are warranted to determine whether these pathways contribute to the anti-inflammatory effects of heteronemin in LPS-stimulated macrophages.

Several marine-derived diterpenes and sesterterpenoids have been reported to possess anti-inflammatory properties [14,26,27,28]. For example, the diterpene excavatolide B suppresses iNOS and COX-2 expression in LPS-stimulated RAW264.7 macrophages [28], whereas the sesterterpenoid phorbaketal A inhibits NO production, iNOS expression, and pro-inflammatory cytokines through suppression of NF-κB signaling in the same cell model [27]. Anti-inflammatory activity has also been reported for other scalarane-type sesterterpenoids in activated immune cells [14,26]. Consistent with these findings, heteronemin, a scalarane-type sesterterpenoid, reduced inflammatory mediators and intracellular ROS in LPS-stimulated macrophages. Given that previous studies on heteronemin have primarily focused on its anticancer activity, the present findings extend its biological relevance to macrophage-mediated inflammatory responses.

From a translational perspective, the observed anti-inflammatory and ROS-modulating activities of heteronemin provide a rationale for further investigation. However, its concentration-dependent cytotoxicity represents an important consideration for future development. Therefore, careful characterization of its therapeutic window and safety profile will be necessary to assess the translational potential of heteronemin and structurally optimized derivatives.

Several limitations should be considered when interpreting the present findings. First, the study was conducted using the RAW264.7 macrophage cell line; therefore, validation in primary macrophages and in vivo models of inflammation is warranted to establish the broader biological relevance of these findings. Second, the absence of an established anti-inflammatory positive control precludes direct comparison of the magnitude of heteronemin-mediated activity with that of a reference compound. Future studies incorporating primary cells, in vivo models, and appropriate positive controls will help further establish the anti-inflammatory efficacy and biological relevance of heteronemin and provide a basis for evaluating its potential for further development.

## 4. Materials and Methods

### 4.1. Preparation and Identification of Heteronemin

Heteronemin was obtained from our previous chemical investigation of the marine sponge *Hyrtios erectus* collected from Bohol Island, Philippines [15]. As detailed in our preceding report, the sequential chromatographic purification of the sponge extract—including solvent partitioning, silica gel chromatography, size-exclusion chromatography, and reversed-phase HPLC—yielded 18 scalarane sesterterpenoids. Among these, heteronemin was selected for the current biological evaluation. Its chemical structure was unambiguously confirmed using ^1^H and ^13^C nuclear magnetic resonance spectroscopy and high-resolution electrospray ionization mass spectrometry (Table S1, Figures S1–S3).

### 4.2. Cell Culture and Cell Viability Assay

RAW264.7 murine macrophage cells were obtained from the Korean Cell Line Bank (KCLB; Seoul, Republic of Korea) and used at passages 4–8 for all experiments. The cells were maintained in Dulbecco’s Modified Eagle Medium (Gibco, Thermo Fisher Scientific, Waltham, MA, USA) supplemented with 10% fetal bovine serum (Gibco, Thermo Fisher Scientific, Waltham, MA, USA) and 1% penicillin–streptomycin (Gibco, Thermo Fisher Scientific, Waltham, MA, USA). Cultures were incubated at 37 °C in a humidified atmosphere containing 5% CO_2_. The LPS concentration (0.5 μg/mL; Sigma-Aldrich, St. Louis, MO, USA; Cat. No. L2654) used for inflammatory stimulation in subsequent experiments was selected based on previous studies demonstrating LPS-induced inflammatory responses in RAW264.7 macrophages [29,30]. Cell viability was assessed using the WST assay. Cells were seeded in 96-well plates (25,000 cells/well) and incubated overnight. Heteronemin (0–20 µM) was then applied for 24 h, followed by the addition of 10 µL of WST-8 reagent (Biomax, Seoul, Republic of Korea). After 1 h of incubation at 37 °C, absorbance was measured at 450 nm using a Synergy HT microplate reader (BioTek Instruments, Winooski, VT, USA).

### 4.3. Measurement of NO Levels

NO levels were assessed by detecting nitrite accumulation using the Griess reaction. Cell culture supernatants (100 µL) were combined with an equal volume of Griess reagent (Promega, Madison, WI, USA), which consisted of 1% sulfanilamide, 0.1% N-(1-naphthyl) ethylenediamine dihydrochloride, and 2% phosphoric acid. After a 15-min incubation at room temperature, absorbance was recorded at 540 nm with a microplate reader, and nitrite concentrations were calculated from a standard sodium nitrite curve.

### 4.4. Measurement of ROS Levels

ROS levels were measured using 2′,7′-dichlorodihydrofluorescein diacetate (H_2_DCFDA; Thermo Fisher Scientific, Waltham, MA, USA). Cells were incubated with 10 µM H_2_DCFDA at 37 °C for 30 min in the dark. After incubation, the fluorescence intensity of the oxidized DCF was immediately quantified using a fluorescence microplate reader (excitation: 488 nm, emission: 535 nm).

### 4.5. RNA Isolation and Real-Time PCR Analysis

Total RNA was purified from cells grown in 60-mm culture dishes using TRIzol™ Reagent (Thermo Fisher Scientific, Waltham, MA, USA) according to the manufacturer’s protocol. RNA quality and concentration were determined using a NanoDrop One spectrophotometer (Thermo Fisher Scientific, Waltham, MA, USA). Subsequently, 1 µg of total RNA was reverse-transcribed into complementary DNA using the PrimeScript™ RT Reagent Kit with gDNA Eraser (Takara, Shiga, Japan; Cat. No. RR047A). Quantitative real-time PCR (qRT-PCR) was performed on a QuantStudio™ 3 Real-Time PCR System (Thermo Fisher Scientific, Waltham, MA, USA) using PowerUp™ SYBR™ Green Master Mix (Thermo Fisher Scientific, Waltham, MA, USA). Glyceraldehyde 3-phosphate dehydrogenase (GAPDH) served as an internal control gene. The PCR amplification program consisted of initial denaturation at 95 °C for 1 min, followed by 40 cycles of denaturation at 95 °C for 15 s and annealing/extension at 60 °C for 1 min. The specificity of the amplified products was confirmed by melt-curve analysis, which showed a single distinct melting peak for each primer set. Relative gene expression levels were calculated using the 2^−ΔΔCt^ method. The primer sequences are shown in Table 1.

### 4.6. Western Blot Analysis

Cells were lysed in radioimmunoprecipitation buffer (Thermo Fisher Scientific) supplemented with a protease/phosphatase inhibitor cocktail (GenDEPOT, Barker, TX, USA) and 1 mM phenylmethylsulfonyl fluoride. Protein concentrations were determined using the Pierce™ BCA Protein Assay Kit (Thermo Fisher Scientific, Waltham, MA, USA). Equal amounts of protein (20 µg per sample) were resolved on NuPAGE™ Bis-Tris 4–12% gradient gels (Thermo Fisher Scientific) and transferred onto 0.45-µm nitrocellulose membranes using the iBlot™ 3 Dry Blotting System. Membranes were blocked with Smart Block™ buffer (Biomax, Guri-si, Republic of Korea) for 10 min at room temperature and incubated overnight at 4 °C with primary antibodies. After three washes with TBST, the membranes were incubated with horseradish peroxidase-conjugated secondary antibodies for 1 h at room temperature. Detailed information on the primary and secondary antibodies, including suppliers, catalog numbers, host species, dilutions, and applications, is provided in Table 2.

Protein bands were visualized using an enhanced chemiluminescence detection kit (Pierce Biotechnology, Rockford, IL, USA), and band intensities were quantified using the iBright™ Analysis Software (version 5.6.0; Thermo Fisher Scientific, Waltham, MA, USA).

### 4.7. Statistical Analysis

Data are presented as the mean ± standard deviation (SD) from three independent experiments. Statistical differences among experimental groups were evaluated using one-way analysis of variance (ANOVA), followed by Dunnett’s multiple-comparisons test. A *p* value < 0.05 was considered statistically significant. All statistical analyses were performed using GraphPad Prism 10 (GraphPad Software, Boston, MA, USA).

## 5. Conclusions

In conclusion, the present study demonstrated that heteronemin, a marine sponge-derived scalarane-type sesterterpenoid, attenuates LPS-induced inflammatory responses in RAW264.7 macrophages under non-cytotoxic conditions. Heteronemin reduced intracellular ROS and NO production and suppressed the expression of inflammatory mediators, including iNOS and COX-2, as well as the phosphorylation of ERK, JNK, and p38. Collectively, these findings suggest that heteronemin has potential as a marine-derived anti-inflammatory compound and support further investigation of its application in anti-inflammatory drug development.

## Figures and Tables

**Table 1:** Primer Sequences Used for Real-Time PCR.

Gene	Primer Sequences
*COX-2*	F 5′-GCGACATACTCAAGCAGGAGCA-3′
	R 5′-AGTGGTAACCGCTCAGGTGTTG-3′
*iNOS*	F 5′-GCTCGCTTTGCCACGGACGA-3′
	R 5′-AAGGCAGCGGGCACATGCAA-3′
*IL-1β*	F 5′-CAGGATGAGGACATGAGCACC-3′
	R 5′-CTCTGCAGACTCAAACTCCAC-3′
*IL-6*	F 5′-GTACTCCAGAAGACCAGAGG-3′
	R 5′-TGCTGGTGACAACCACGGCC-3′
*TNF-α*	F 5′-TTGACCTCAGCGCTGAGTTG-3′
	R 5′-CCTGTAGCCCACGTCGTAGC-3′
*GAPDH*	F 5′-AAGGGTCATCATCTCTGCCC-3′
	R 5′-GTGATGGCATGGACTGTGGT-3′

**Table 2:** Antibodies used for Western blot analysis.

Antibody	Supplier	Catalog No.	Host Species	Dilution	Application
NOS (pan) Antibody	Cell Signaling Technology	2977	Rabbit	1:1000	Western blot
COX-2 Antibody	Cell Signaling Technology	4842	Rabbit	1:1000	Western blot
p-ERK (E-4) Antibody	Santa Cruz Biotechnology	sc-7383	Mouse	1:1000	Western blot
p44/42 MAPK (ERK1/2) (137F5) Rabbit mAb	Cell Signaling Technology	4695	Rabbit	1:1000	Western blot
Phospho-SAPK/JNK (Thr183/Tyr185) Antibody	Cell Signaling Technology	9251	Rabbit	1:1000	Western blot
SAPK/JNK Antibody	Cell Signaling Technology	9252	Rabbit	1:1000	Western blot
Phospho-p38 MAPK (Thr180/Tyr182) (D3F9) Rabbit mAb	Cell Signaling Technology	4511	Rabbit	1:1000	Western blot
p38 MAPK (D13E1) Rabbit mAb	Cell Signaling Technology	8690	Rabbit	1:1000	Western blot
GAPDH (14C10) Rabbit mAb	Cell Signaling Technology	2118	Rabbit	1:1000	Western blot
Anti-rabbit IgG, HRP-linked Antibody	Cell Signaling Technology	7074	Goat	1:5000	Western blot
Anti-mouse IgG, HRP-linked Antibody	Cell Signaling Technology	7076	Horse	1:5000	Western blot

## Data Availability

Data from this study are available from the corresponding authors upon reasonable request.

## Supplementary Materials

The following supporting information can be downloaded at: https://www.mdpi.com/article/10.3390/md24100351/s1, Table S1: ^1^H and ^13^C NMR data for heteronemin compared with previously reported data [31]; Figure S1: ^1^H NMR spectrum of heteronemin in CDCL_3_ (600 Hz); Figure S2: ^13^C NMR spectrum of heteronemin in CDCL_3_ (150 Hz); Figure S3: HRESIMS data of heteronemin (*m*/*z* 511.3034 [M + Na]+; calcd for C29H44O4Na+ 511.3030).

## Abbreviations

The following abbreviations are used in this manuscript:

LPS	lipopolysaccharide
COX-2	cyclooxygenase-2
NO	nitric oxide
iNOS	inducible nitric oxide synthase
ROS	reactive oxygen species
H_2_DCFDA	2′,7′-dichlorodihydrofluorescein diacetate
IL-1β	interleukin-1 beta
IL-6	interleukin-6
TNF-α	tumor necrosis factor
