# Supplementary material for: Heteronemin Attenuates LPS-Induced Oxidative Stress and Inflammatory Responses in RAW264.7 Macrophages
Source: Mar Drugs. 2026 Oct 6;24(10):351. doi: 10.3390/md24100351 (PMC13705918; doi:10.3390/md24100351)
Supplement: Supplementary file 1 [file marinedrugs-24-00351-s001.zip › marinedrugs-4575750-supplementary.pdf]

Supplementary Materials

Heteronemin Attenuates LPS-Induced Oxidative Stress and Inflammatory Responses in RAW264.7 Macrophages

Su-Jin Lee [1], Long Hoang To [2,3], Chulhong Oh [1,3], Gun-Hoo Park [1,3,*] and Yeon-Ju Lee [2,3,*]

1 Korea Institute of Ocean Science and Technology, 2670, Iljudong-ro, Gujwa-eup, Jeju-si 63349, Republic of Korea
2 Korea Institute of Ocean Science and Technology, 385, Haeyang-ro, Yeongdo-gu, Busan Metropolitan City 49111, Republic of Korea; longhto@kiost.ac.kr (L.H.T.)
3 Department of Marine Technology and Convergence Engineering, University of Science and Technology, Daejeon 34113, Republic of Korea
* Correspondence: gunhoopark@kiost.ac.kr (G.-H.P.); yjlee@kiost.ac.kr (Y.-J. L.)

Table S1. ^{1}H and ^{13}C NMR data for compound **1** compared with previously reported data[31]

Position	Observed data **1** ($CDCl_3$)		Reported data of **1** ($CDCl_3$)	
	δ_C	δ_H, mult (J in Hz)	δ_C	δ_H, mult (J in Hz)
1_{ax}	39.9	0.78, ovl[a]	39.9	0.78, m
1_{eq}		1.69, ovl[a]		1.69, m
2_{ax}	18.2	1.34, ovl[a]	18.2	1.34, m
2_{eq}		1.37, ovl[a]		1.37, m
3_{ax}	42.2	1.12, ovl[a]	42.2	1.12, m
3_{eq}		1.37, ovl[a]		1.37, m
4	33.2	-	33.2	-
5	56.5	0.55, ovl[a]	56.5	0.55, m
6_{ax}	18.6	1.42, ovl[a]	18.6	1.42, m
6_{eq}		1.62, ovl[a]		1.62, m
7_{ax}	41.8	0.91, ovl[a]	41.8	0.91, m
7_{eq}		1.74, ovl[a]		1.74, m
8	37.4	-	37.4	-
9	58.7	0.86, ovl[a]	58.7	0.86, m
10	38.0	-	38.0	-
11_{ax}	27.2	1.46, ovl[a]	27.2	1.46, m
11_{eq}		1.71, ovl[a]		1.71, m
12	80.5	3.44, dd, J = 3.5, 11.4	80.5	3.45, d, J = 11.4
13	42.7	-	42.7	-
14	54.6	0.94, ovl[a]	54.6	0.94, m
15_{ax}	28.0		28.0	
15_{eq}		2.06, ddd, J = 2.4, 6.1, 12.1		2.06, ddd, J = 2.4, 6.1, 12.1
16	69.3	5.35, dddd, J = 1.8, 1.8, 6.1,	69.3	5.37, dddd, J = 1.8, 1.8, 6.1,

		10.4		10.4
17	114.7	-	114.7	-
18	64.2	2.43, s	64.2	2.43, brs
19	101.7	6.76, d, *J* = 1.4	101.7	6.78, d, *J* = 1.4
20	135.3	6.15, t, *J* = 1.4	135.3	6.17, t, *J* = 1.8
21	21.4	0.78, s	21.4	0.79, s
22	33.2	0.82, s	33.2	0.83, s
23	16.3	0.81, s	16.3	0.82, s
24	17.3	0.83, s	17.3	0.84, s
25	8.7	0.89, s	8.7	0.90, s
26	171.3	-	171.3	-
27	21.2	2.10, s	21.2	-
28	170.1	-	170.1	2.11, s
29	21.1	2.09, s	21.1	2.11, s

[a]Overlapped with other signals.

31 Kazlauskas, R.; Murphy, P. T.; Quinn, R. J.; Wells, R. J. Heteronemin, a New Scalarin Type Sesterterpene from the Sponge Heteronema Erecta. *Tetrahedron Lett.* **1976**, *17* (30), 2631–2634. https://doi.org/10.1016/S0040-4039(00)91753-2

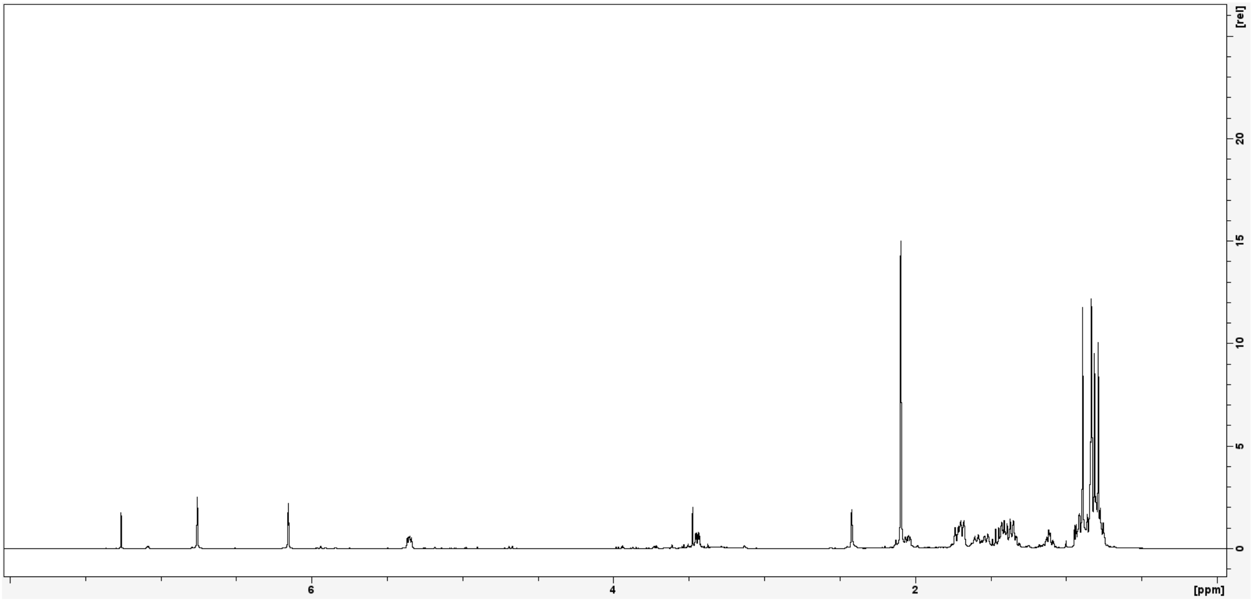

Figure S1. ^{1}H NMR spectrum of heteronemin in $CDCl_3$ (600 Hz)

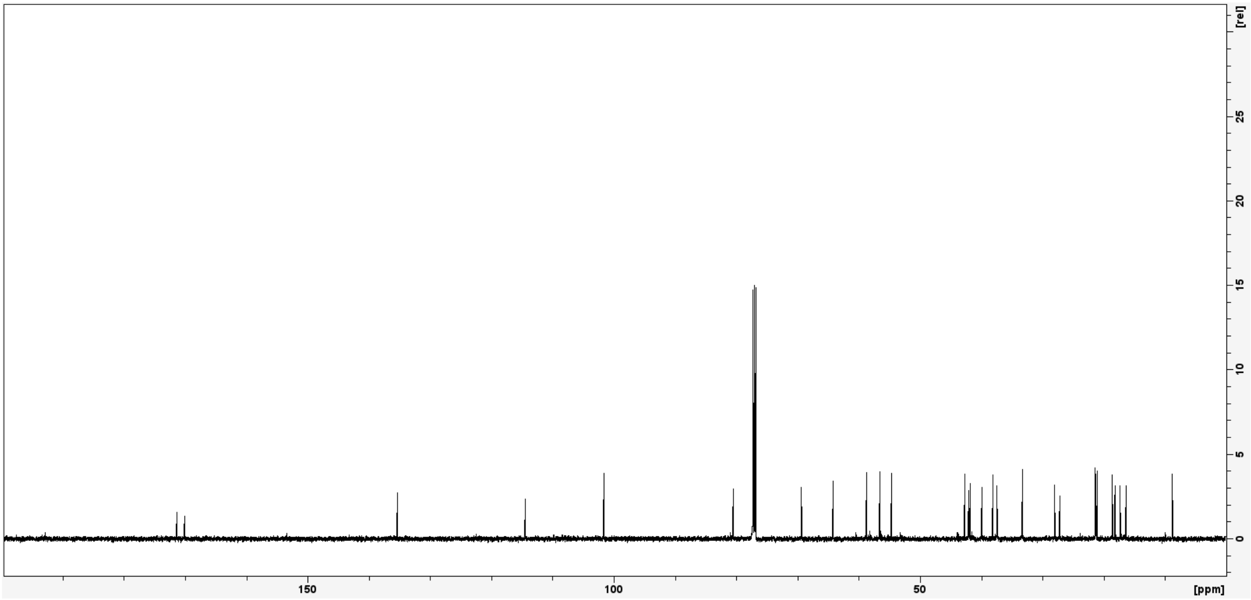

Figure S2. ^{13}C NMR spectrum of heteronemin in $CDCl_3$ (150 Hz)

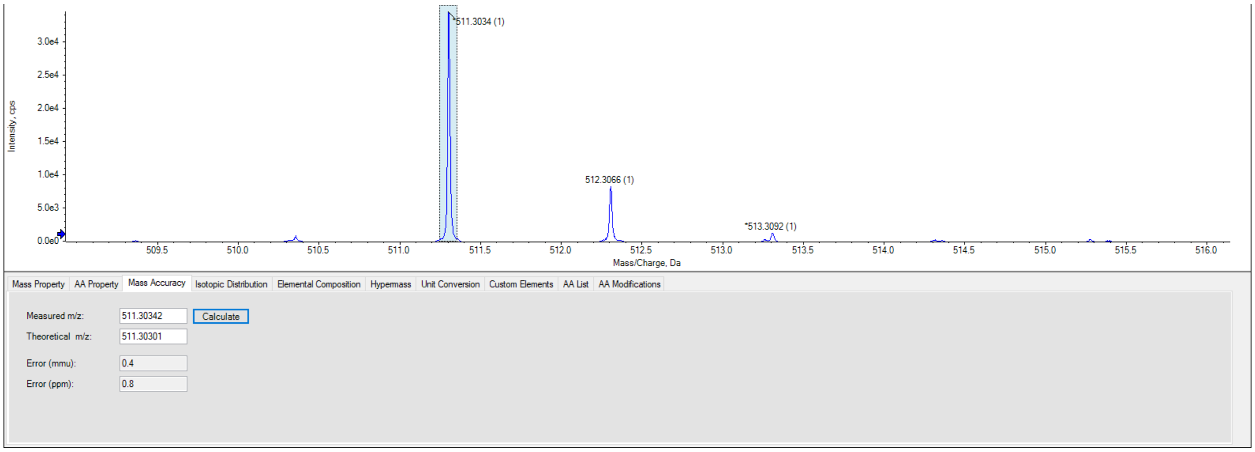

Figure S3. HRESIMS data of heteronemin

(*m*/*z* 511.3034 $[M + Na]^+$; calcd for $C_{29}H_{44}O_4Na^+$ 511.3030)
